# Haustorium initiation in the obligate parasitic plant *Phelipanche ramosa* involves a host-exudated cytokinin signal

**DOI:** 10.1093/jxb/erx359

**Published:** 2017-10-21

**Authors:** Vincent Goyet, Estelle Billard, Jean-Bernard Pouvreau, Marc-Marie Lechat, Sandra Pelletier, Muriel Bahut, Fabrice Monteau, Lukáš Spíchal, Philippe Delavault, Grégory Montiel, Philippe Simier

**Affiliations:** 1 Université de Nantes, Laboratoire de Biologie et Pathologie Végétales, EA 1157, SFR 4207 QUASAV, UFR Sciences et Techniques, 44322 Nantes, France; 2 IRHS UMR1345, INRA, AGROCAMPUS-Ouest, Université d’Angers, SFR 4207 QUASAV, 42 rue Georges Morel, 49071 Beaucouzé cedex, France; 3 Plateau Technique Mutualisé ANAN, SFR 4207 QUASAV, 42 rue Georges Morel, 49071 Beaucouzé, France; 4 ONIRIS, USC 2013, LABERCA, Atlanpole-La Chantrerie, BP 50707, 44307 Nantes, France; 5 Department of Chemical Biology and Genetics, Centre of the Region Haná for Biotechnological and Agricultural Research, Faculty of Science, Palacký University, Olomouc, CZ-78371, Czech Republic

**Keywords:** *Brassica napus*, broomrape, cytokinins, haustorium, microarray, parasitic plant, *Phelipanche ramosa*, root development, root exudates

## Abstract

The heterotrophic lifestyle of parasitic plants relies on the development of the haustorium, a specific infectious organ required for attachment to host roots. While haustorium development is initiated upon chemodetection of host-derived molecules in hemiparasitic plants, the induction of haustorium formation remains largely unknown in holoparasitic species such as *Phelipanche ramosa*. This work demonstrates that the root exudates of the host plant *Brassica napus* contain allelochemicals displaying haustorium-inducing activity on *P. ramosa* germinating seeds, which increases the parasite aggressiveness. A *de novo* assembled transcriptome and microarray approach with *P. ramosa* during early haustorium formation upon treatment with *B. napus* root exudates allowed the identification of differentially expressed genes involved in hormone signaling. Bioassays using exogenous cytokinins and the specific cytokinin receptor inhibitor PI-55 showed that cytokinins induced haustorium formation and increased parasite aggressiveness. Root exudates triggered the expression of cytokinin-responsive genes during early haustorium development in germinated seeds, and bio-guided UPLC-ESI(+)-/MS/MS analysis showed that these exudates contain a cytokinin with dihydrozeatin characteristics. These results suggest that cytokinins constitutively exudated from host roots play a major role in haustorium formation and aggressiveness in *P. ramosa*.

## Teaser

The formation of the haustorium in holoparasitic plants as demonstrated in *Phelipanche ramosa* is initiated through perception of a host-exudated cytokinin and a cytokinin signaling response.

## Introduction

Some terrestrial plant species have developed unique features in the course of evolution, leading them to adopt a heterotrophic lifestyle. Plant parasitism is thought to have evolved independently 12–13 times in angiosperms, allowing the occurrence of a diversity of parasitic behavior ranging from opportunistic facultative parasitism to obligatory parasitism (Westwood *et al.*, 2010). The transition from autotrophy to parasitism lies in the development of a multicellular specialized infectious organ, the haustorium, which allows adhesion to and penetration of host tissues by the parasitic plant and the establishment of vascular connections between the host and the parasite (Yoshida *et al.*, 2016).

Most plants in the Orobanchaceae family are root parasitic organisms, including the facultative parasites *Triphysaria versicolor* and *Phtheirospermum japonicum*, and the obligatory parasites *Striga* sp. (witchweeds), and *Orobanche* and *Phelipanche* sp. (broomrapes). Facultative parasites can grow to maturity without host connections, although in the vicinity of a host root, they can develop lateral haustoria to connect to the host vascular system (Albrecht *et al.*, 1999). Lateral haustoria develop at the root elongation zone without interfering with the meristematic activity of the root apex, allowing the root growth to resume after the initiation of haustorium formation (Matvienko *et al.*, 2001*a*). Obligatory parasites develop terminal haustoria at the apex of the radicle of germinated seeds which cause a complete root growth arrest and a redirection of cellular extension from longitudinal to radial (Joel and Losner-Goshen, 1994; Hood *et al.*, 1998). In chlorophyllous species (hemiparasites) including witchweeds and facultative parasites, the development of early haustorial structures (EHSs) starts with the swelling of the root tip due to the redirection of cell division. Concomitantly, a polar outgrowth of epidermal cells is observed which give rise to haustorial hairs that serve as structures for adhesion to the host (Baird and Riopel, 1983; Hood *et al.*, 1998; Bandaranayake and Yoder, 2013). In achlorophyllous species (holoparasites) such as *Orobanche cumana* and *Phelipanche aegyptiaca*, the early haustorium development is also revealed by a swelling of the root tip, caused by the extension of the apical cells and a polar outgrowth of epidermal cells, but it gives rise to short cell extensions termed papillae (Joel and Losner-Goshen, 1994; Fernández-Aparicio *et al.*, 2016). Both haustorial hairs and papillae produce adhesive molecules meant to anchor the haustorium to the host root (Baird and Riopel, 1985; Joel and Losner-Goshen, 1994).

EHS formation is triggered after a host-derived chemical stimulation by so-called haustorium-inducing factors (HIFs), which are mainly phenolic acids (Albrecht *et al.*, 1999). Among them, 2,6-dimethoxy-1,4-benzoquinone (DMBQ) was isolated from abraded *Sorghum* roots and shown to trigger haustorium formation in *T. versicolor* and *Striga hermonthica* (Lynn *et al.*, 1981; Chang and Lynn, 1986). DMBQ is released by oxidation of host cell wall compounds upon host peroxidase activity triggered by hydrogen peroxide production at the tip of the *Striga* radicle (Keyes *et al.*, 2000). Such a model explains how the parasite extracts HIFs and develops an haustorium only upon close contact with the host root (Keyes *et al.*, 2007). DMBQ recognition involves a modification of redox potential, in which semiquinone intermediates are formed to trigger a redox-sensitive signal transduction pathway (Keyes *et al.*, 2000). This redox cycling involves two quinone oxidoreductase-encoding genes (*TvQR1* and *TvQR2*) which are transcriptionally up-regulated in *T. versicolor* roots in response to DMBQ (Bandaranayake *et al.*, 2010; Ishida *et al.*, 2017). Treatments with phytohormones also trigger EHS formation (i.e. haustorial hairs) (O’Malley and Lynn, 2000), but with differences in the degree and size of the swelling and in the precise and synchronous location of the haustorial hairs with respect to the root apex (Tomilov *et al.*, 2005). Nevertheless, indole acetic acid (IAA) treatment enhances haustorial hair formation in *T. versicolor* when low DMBQ concentrations are applied. Ishida *et al.* (2016) showed that the auxin biosynthesis gene *YUC3* encoding a flavin monooxygenase is up-regulated in haustorial cells of *P. japonicum*. Overexpression of *YUC3* in *P. japonicum* roots triggers haustorium formation, and IAA levels increase at the haustorium formation site after DMBQ treatment. These results suggest that the auxin biosynthetic and regulatory pathways may be recruited in these facultative parasites during EHS formation.

Although no known host-derived HIF is active on holoparasites, recent findings showed that the mycotoxins sphaeropsidones may trigger EHS formation in *O. cumana* and *O. crenata* (Fernández-Aparicio *et al.*, 2016). These molecules are not related in structure to DMBQ, which suggests that other receptors and signaling pathways leading to EHS formation in broomrapes may exist. Although recent transcriptomic studies have unraveled the existence of core parasitism genes expressed in response to DMBQ in *S. hermonthica*, *T. versicolor*, and *P. aegyptiaca* (Yang *et al.*, 2015), and strengthened the involvement of hormone signaling in EHS development in *P. japonicum* and *Santalum album* (Zhang *et al.*, 2015; Ishida *et al.*, 2016), the mechanisms leading to haustorium formation in the holoparasite *P. aegyptiaca* remain largely unknown.

Here, using transcriptomic and biochemical approaches, we examined the induction of haustorium formation in the holoparasitic species *P. ramosa* in response to host plant root exudates (*Brassica napus*), and demonstrated that this event is triggered by a cytokinin (CK) signal.

## Materials and methods

### Plant material and growth conditions

*Phelipanche ramosa* (L.) Pomel seeds were collected in 2014 from mature broomrape flowering spikes in an oilseed rape field at Saint Martin de Fraigneau (France) and stored at 25 °C in the dark before use. The highly susceptible *B. napus* cultivar ES Aliénor (EURALIS, Lescar, France) was chosen for co-cultivation experiments in Petri dishes and exudation assays. *S*eeds were surface-sterilized using 2.4% sodium hypochlorite (Lechat *et al.*, 2012). *Phelipanche ramosa* seeds were then suspended (10 mg ml^–1^) in 1 mM HEPES, pH 7.5, 0.1% PPM (incubation medium) and placed at 21 °C in the dark for 7 d (conditioning period; Lechat *et al.*, 2015).

*Brassica napus* seeds were placed on a plate between two moistened filter papers and incubated at 21 °C in a growth chamber (16 h light, 8 h dark) for 7 d. Two hundred plantlets were then distributed into 20 drained plastic pots [700 ml, 9 × 11 × 12 cm (W×L×H), Nicoplast] nested in undrained pots and filled with glass beads (Ø 3 mm). Plantlets were first watered with 100 ml of 50% Coïc medium pH 6.8 (Coic and Lesaint, 1975) once a week and then every 2 d from week 3 on. Culture medium (root exudates) from each drained pot was recovered and pooled at the end of weeks 3 and 4 (weeks with exudates displaying the highest EHS induction activity).

### Bioassays for haustorial induction

After the conditioning period, the incubation medium was removed and *P. ramosa* seeds were rinsed three times with sterile distilled water and suspended (2.5 mg ml^–1^) in germination medium [0.5 mM HEPES, pH 7.5, 0.05% PPM, *rac*-GR24 10^–7^ M (synthetic germination stimulant, kindly provided by Dr B. Zwanenburg)]. A 100 µl aliquot of GR24-treated *P. ramosa* seeds (~25 seeds) was then distributed in each well in 96-well plates (Cell Culture Multiwell Plate Cellstar; Greiner Bio-One) and placed to germinate at 21 °C in the dark for 4 d.

The germination solution was then removed from the wells and seeds were suspended in 100 µl of test solutions. Test solutions for EHS induction assays contain 0.5 mM HEPES, pH 7.5, 0.05% PPM (final concentration) and various concentrations of commercial standards and organic or aqueous root exudates from *B. napus* (Supplementary Table S1 at *JXB* online). Induction of EHS was monitored 3 d after treatments.

### Confocal laser and light microscopy

Observations of haustorium formation at the apex of *P. ramosa* germinated seeds were conducted using a confocal laser scanning microscope (MCBL Nikon A1). Germinated seeds were treated or not with *B. napus* root exudates for 72 h and then stained in acridine orange (0.02%) for 5 min and rinsed three times with water. The excitation and emission wavelengths were set at 488 nm and 500–530 nm, respectively.

Germinated seeds were fixed and embedded in LRW resin (London Resin White acrylic, Sigma Aldrich) (Chateigner-Boutin *et al.*, 2014). Sections of 1.5 µm thickness were prepared using a Leica EM UC7 ultramicrotome, stained using toluidine blue (Toluidine blue 1%, Na_2_CO_3_ 2.5%) for 5 min, and rinsed with distilled water, then observed using a Nikon AZ100 macroscope.

### Aggressiveness bioassay

For co-cultivation experiments, 7-day-old *B. napus* plantlets were transferred onto filter paper each covered with 20 mg of germinated *P. ramosa* seeds previously treated or not for 2 d with root exudates and placed in cut square plates (120 × 120 × 17 mm, Greiner, France) containing a uniform layer of rockwool moisturized with 50 ml of Coïc medium. Five plantlets per plate and five plates per condition were used. Plates were sealed and incubated vertically at 21 °C in a growth chamber (16 h light, 8 h dark, 70% humidity) for 17 d and supplied every 2 d with 10 ml of Coïc medium. The development of a functional haustorium was monitored daily using a phloem-mobile fluorescent dye (6-Carboxyfluorescein, Sigma Aldrich, 10 mM, pH 6.3). Droplets of carboxyfluorescein were applied on abraded host leaves, and fluorescent tubercles were observed using a fluorescence binocular equipped with a GFP2 filter: 480/40 nm excitation; 510 nm emission (Leica), showing functional phloem connections between the host and parasite within the haustorium (Péron *et al.*, 2017).

### Total RNA extraction

RNA was extracted using a Nucleospin RNA Plant kit (Macherey Nagel) according to the manufacturer’s instruction. Genomic DNA was removed from RNA samples by treatment with 6.8 U of RNase-free DNase I (Qiagen, Cat. No 79254) followed by a clean-up step with an RNA clean up XS spin column (Macherey Nagel) according to the manufacturer’s instruction. RNA was quantified using a NanoDrop ND-1000 (NanoDrop Technologies), and RNA quality was assessed with an Agilent 2100 Bioanalyzer.

### cDNA sequencing and *de novo* transcriptome assembly

Total RNAs were extracted from *P. ramosa* seeds imbibed for 24 h, seeds conditioned for 7 d, germinated seeds (72 h after GR24 treatment), young tubercles, ‘spider’ stage tubercles, young flowering stems, and mature flowering stems. A Roche 454 normalized library was constructed with mRNAs isolated from each sample and mixed in equal proportion, and single read sequences were obtained with Roche GS-FLX+ 454 pyrosequencing systems equipped with on-instrument Data Analysis Software Modules v.2.3 (Life Science). cDNA library construction and sequencing were performed by Eurofins Genomics Company (Ebersberg, Germany).

For *de novo* transcriptome assembly, sequences were treated using the Mira sequence assembler Version 3.4 by Eurofins Genomics Company. Blast annotation of the translated sequences (BlastX) was performed using the BioEdit software v7.2.5 against *Arabidopsis thaliana* (TAIR 10) and *Solanum lycopersicum* (SlGDB, http://www.plantgdb.org) libraries. The contigs were then assigned to the PlantTribes 2.0 orthogroups (Wall *et al.*, 2008). Gene Ontology (GO) terms were assigned for each unigene based on GO slim terms using the PANTHER algorithm (http://www.pantherdb.org) (Mi *et al.*, 2016).

### Microarray analysis

Contigs obtained from *de novo* transcriptome assembly were used to design the Agilent-069670 Phelipanche_v1 chip. It was *in situ* synthesized by Agilent (see the manufacturer’s web site at http://www.agilent.com/) and contained 105 086 oligoprobes of 60-mers as follows: 52 543 predicted sense and 52 543 complementary antisense sequences corresponding to the predicted coding genes. All probes were designed within the CDS and with a positive bias toward the 3′ end of the genes, and fixed on Agilent 4 X 180K microarray slides (Gene Expression Omnibus under the accession number GPL23512).

Total RNA was extracted from 200 mg of germinated seeds treated or not with crude *B. napus* root exudates for 12 h and 24 h then deep frozen in liquid nitrogen. mRNA was amplified, labeled as described in Celton *et al.* (2014), and co-hybridized according to the Agilent two-color microarray-based gene expression analysis protocol.

For each comparison, two biological replicates were analyzed in dye-switch design as described in Depuydt *et al.* (2009). All statistical analyses were performed using the R language (R Development Core Team, 2009) as described in Celton *et al.* (2014). Transcriptome data have been submitted to the Gene Expression Omnibus as Series GSE99377. GO term enrichment of differentially expressed genes (DEGs) was carried out using the AGRIGO algorithm (http://bioinfo.cau.edu.cn/agriGO) (Du *et al.*, 2010) against the GO assignment of *P. ramosa de novo* transcriptome assembly using Fisher’s exact test corrected as described in Benjamini and Hochberg (1995) [false discovery rate (FDR) <0.05].

### Quantitative real-time PCR (RT-qPCR) analysis

cDNA was synthesized from 0.5 μg of total RNA using SuperMIX qScript cDNA (Quanta) according to the manufacturer’s instructions. cDNA was diluted 20 times and used in 25 µl RT-PCR experiments with 12.5 µl of PerfeCTa SYBR Green SuperMix (Quanta) and 0.3 mM of each primer, carried out on an Applied Biosystems 7300 real-time PCR system (Applied Biosystems) as described in Lechat *et al.* (2015) using the amplicon of the constitutive 18S, which showed low cycle threshold variation (SD <0.5 cycle threshold), as an internal control to normalize all the data. The gene-specific primers used for each amplification are presented in Supplementary Table S2. A control experiment without cDNA was included for each PCR mix.

### Bioassay-guided reversed phase (RP)-HPLC purification of HIFs

Two liters of crude *B. napus* root exudates were loaded on a Strata-X 33µm Polymeric Solid Phase Extraction column (SPE, Phenomenex) and then washed with 4 ml of distilled water. Flow-through and washing volumes were pooled (FT). Compounds eluted with 4 ml of acetonitrile (ACN) were further evaporated using an R II Rotavapor (Buchi) and suspended in 200 µl of 50% ACN (SPE extract).

The SPE extract was further fractionated by using a LaChrom Elite HPLC System (Hitachi) equipped with an L-2100 pump, L-2200 Autosampler, L-2400 UV Detector, and column thermostat Jetstream 2 Plus. A 90 µl aliquot of SPE extract was loaded on a Kromasil C18 guard column (5 µm, 250 × 4.6 mm, Sigma-Aldrich). Chromatographic conditions were: flow rate of 0.5 ml min^−1^; column temperature of 30 °C; and the following binary gradient: 0 min, 95:5; 7 min, 95:5; 28 min, 0:100; 39 min, 0:100 (time, water–0.1% acetic acid:ACN–0.1% acetic acid). Absorbance was monitored at 260 nm. Fractions 0.5 ml to 2.5 ml of the purified exudate were collected from 0 to 39 min and tested in an haustorium induction assay.

### Detection and quantification of CKns by UPLC-ESI (+)-MS/MS in root exudates

CK identification in the fractionated exudates was carried out by UPLC-ESI(+)-MS/MS according to Svačinová *et al.* (2012) with some modifications. An Acquity UPLC System (Waters) linked to a triple quadrupole mass spectrometer Xevo™ TQ-S (Waters MS Technologies) equipped with an electrospray interface (ESI) and the unique performance of a collision cell (ScanWave) was used. MS data were processed by MassLynx™ software with the TargetLynx™ program (version 4.2, Waters).

A 2 µl aliquot of samples (SPE extract or fractions) was injected onto a reversed-phase column (Acquity UPLC BEH C18, 1.7 μm, 2.1 × 100 mm, Waters). The flow rate was fixed at 0.4 ml min^−1^; column temperature was 40 °C; sample temperature was 4 °C; and the following binary gradient (A, water–0.5% formic acid; B, methanol–0.5% formic acid) was applied: 0 min, 95:5 (A:B); 4.0 min, 95:5; 9.0 min, 50:50; 10.0 min, 0:100; 1.0 min, 0:100; 13.0 min, 95:5; 15.0 min, 95:0. Selective reaction monitoring mode using mass-to-charge (*m/z*) transitions of precursor and product ions was optimized: capillary voltage 3.0 kV; source/desolvation gas temperature 150/400 °C; cone/desolvation gas flow rates 150/650 l h^−1^.

## Results

### *Brassica napus* root exudates induce EHSs at the apex of the *P. ramosa* radicle

**Fig. 1.**
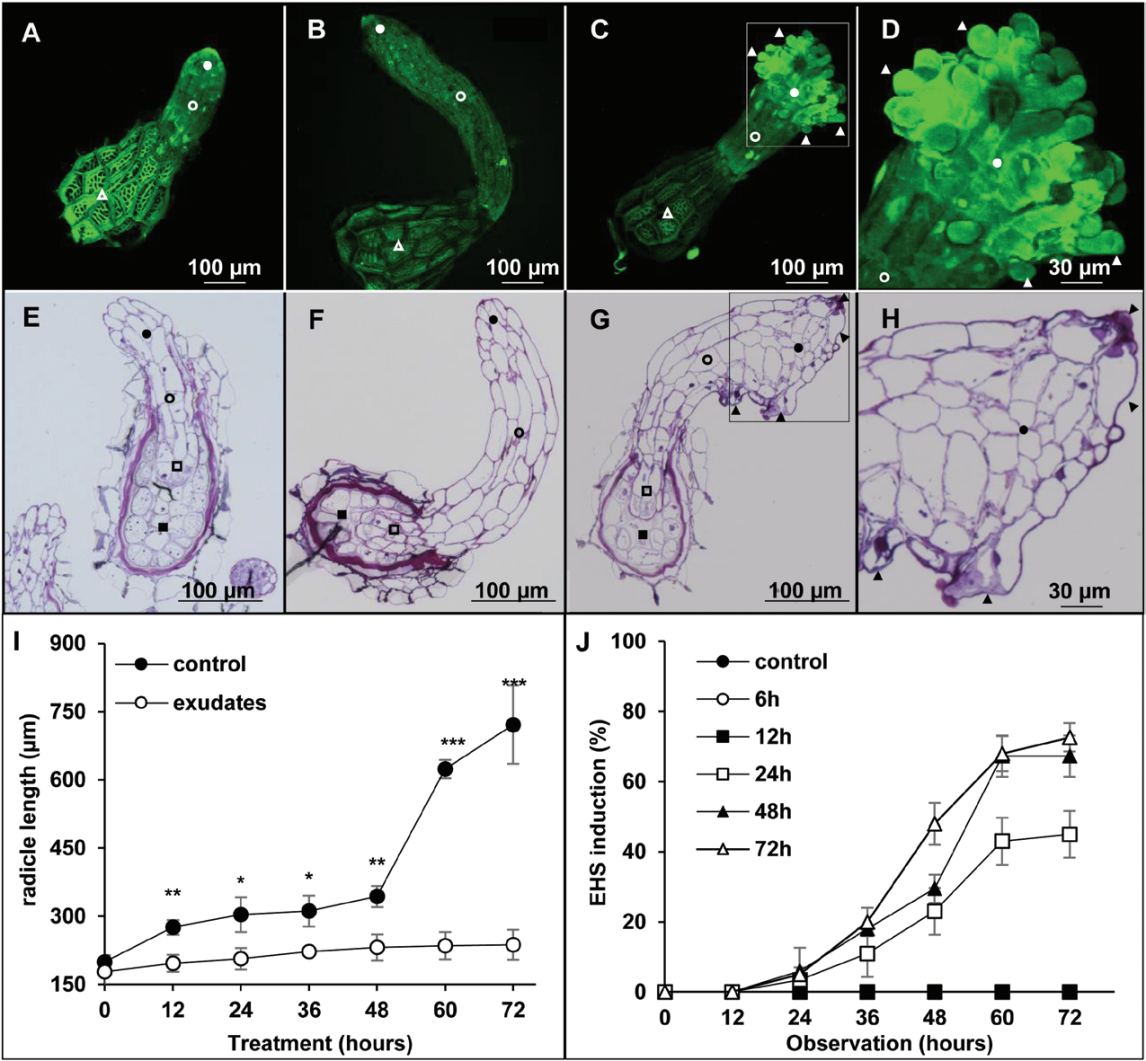
*Brassica napus* root exudates can trigger the formation of early haustorial structures in *P. ramosa*. (A–D) Confocal laser scanning microscope observation of *P. ramosa* germinated seeds stained with acridine orange. (E–H) Light microscope observation of thin sections of *P. ramosa* germinated seeds stained with toluidine blue. Open squares, embryo; filled squares, albumen; open circles, radicle; filled circles, apex; open triangles, testa; filled triangles, papillae. (A, E) Seeds before treatment. (B, F) Seeds incubated for 72 h in control buffer. (C, G) Seeds incubated for 72 h in half-diluted *B. napus* root exudates. Sale bars=100 µm. (D, H) Close-up view of the root apex of seeds incubated for 72 h in *B. napus* root exudates. Scale bars=30 µm. (I) Assessment of radicle length of *P. ramosa* germinated seeds upon treatment with *B. napus* root exudates during 72 h. Exudates were half diluted in buffer solution (0.5 mM HEPES, 0.05% PPM). The control condition corresponds to a treatment with the buffer solution alone. Means are values ±SE (*n*=6). Means annotated with asterisks are significantly different from the control points (*t*-test, *P<0.05, **P<0.01, and ***P<0.001). (J) Time course assessment of EHS development upon continuous treatment with *B. napus* root exudates for 6, 12, 24, 48, and 72 h, respectively. At the end of each treatment time, germinated seeds were rinsed, and exudates were discarded and replaced by buffer solution. Means are values ±SE (*n*=6).

By choosing *B. napus* as a major host for *P. ramosa*, we tested if the signal allowing the induction of haustorium formation is of allelochemical nature and present in the root exudates. Germinated seeds (96 h post 10^−7^ M GR24 treatment, T_0_ seeds) were treated or not with *B. napus* root exudates during 72 h. While T_0_ seeds and untreated seeds displayed radicles with a thin and regular conical apex (Fig. 1A, B, E, F), treated seeds displayed a larger apex with irregular lobes and larger cells (Fig. 1C, G). Moreover, a complete root growth arrest was observed in treated germinated seeds in comparison with the untreated radicles (Fig. 1I). This suggests that root exudates trigger a change in cell expansion and root elongation. In addition, papillae were observed in the crown at the apex of treated radicles, each arising from single epidermis cells (Fig. 1C, D, G, H). EHSs are characterized by the swelling of the root apex, shorter roots, and outgrowth of epidermal cells (formation of papillae).

A time course treatment was used to determine the kinetics of EHS formation as well as the minimum treatment time required. Untreated seeds and seeds treated for 6 h and 12 h did not show any EHS formation in the course of the experiment, whereas seeds treated for 24, 48, and 72 h all displayed a significant EHS formation at 36 h and reached up to 72% of radicles displaying EHSs at 72 h (Fig. 1J). A dose–response pattern was observed using diluted extracts (Supplementary Fig. S1).

Taken together, these results (root growth arrest and EHS formation) suggest that *B. napus* root exudates contain molecules acting as HIFs.

### Induction of EHSs enhances aggressiveness of *P. ramosa* germinated seeds

**Fig. 2.**
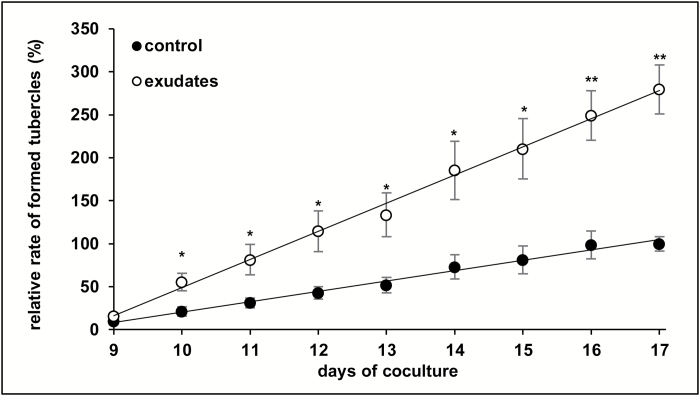
Induction of EHSs at the apex of *P. ramosa* germinated seeds enhances parasite aggressiveness. A 20 mg aliquot of *P. ramosa* germinated seeds was incubated for 48 h in *B. napus* root exudates or control solution before placing them in contact with *B*. *napus* roots. Five plantlets per plate and five plates per condition were used. The number of developing tubercles was assessed using the phloem-mobile dye 6-carboxyfluorescein and the results were expressed as a percentage of the maximum number of tubercles observed in control conditions. Exudates were half diluted in buffer solution (0.5 mM HEPES, 0.05% PPM). The control condition corresponds to a treatment with the buffer solution alone. Means are values ±SE (*n*=5 plates). Means annotated with asterisks are significantly different from the control points (*t*-test, *P<0.05 and **P<0.01).

Co-cultivation assays were used in order to demonstrate the importance of the observed EHSs for the parasite aggressiveness. Germinated *P. ramosa* seeds were treated or not for 48 h with *B. napus* root exudates before being put in contact with roots of *B. napus* plantlets. The number of tubercles with vascular connections reflecting the development of functional haustoria was assessed using the phloem tracer, 6-carboxyfluorescein. The results were expressed as a percentage of the maximum number of tubercles observed in control conditions (no treatment with *B. napus* root exudates). The number of tubercles in treated conditions was already significantly higher (55 ± 10%) than in untreated conditions (21 ± 6%) from the 10th day on and reached 280 ± 28% at the end of the experiment (Fig. 2), indicating that pre-inducing the development of EHSs clearly increased the aggressiveness of the parasite.

### High-throughput sequencing and *de novo* assembly of the *P. ramosa* transcriptome

To obtain *de novo* assembly of the *P. ramosa* transcriptome, a GS-FLX+ 454 pyrosequencing approach (Life Science) was used. Total RNAs were extracted from various developmental stages from seeds to mature flowering shoots. A normalized cDNA library was constructed from mRNAs isolated from each sample and mixed in equal proportion. A total of 1 151 288 reads with an average length of 437 bp and a total length of 503 Mbp were generated. The assembly yielded 58 Mbp distributed in 53 511 unigenes (non-redundant sequences; Supplementary Table S3). The unigenes had an average size of 952 bp and an N50 contig length of 1071 (L50=20 080).

The coverage rate of the *P. ramosa* transcriptome was assessed by examining the proportion of conserved orthologous genes in the assembly data set. Two sets of conserved genes were used, namely the UCOs containing 357 single-copy genes highly conserved in *A. thaliana*, humans, mice, yeast, fruit flies, and *Caenorhabditis elegans*, and APVO sequences containing 959 single-copy genes from *A. thaliana*, *Populus trichocarpa*, *Vitis vinifera*, and *Oryza sativa* plant genomes (Wall *et al.*, 2008; Duarte *et al.*, 2010): 98% of UCOs (with a threshold blast e-value of 1 × 10^10^) and 82% of APVO sequences were detected in *P. ramosa* transcriptome assembly, indicating the high coverage rates of *P. ramosa* transcripts (Supplementary Table S3).

**Fig. 3.**
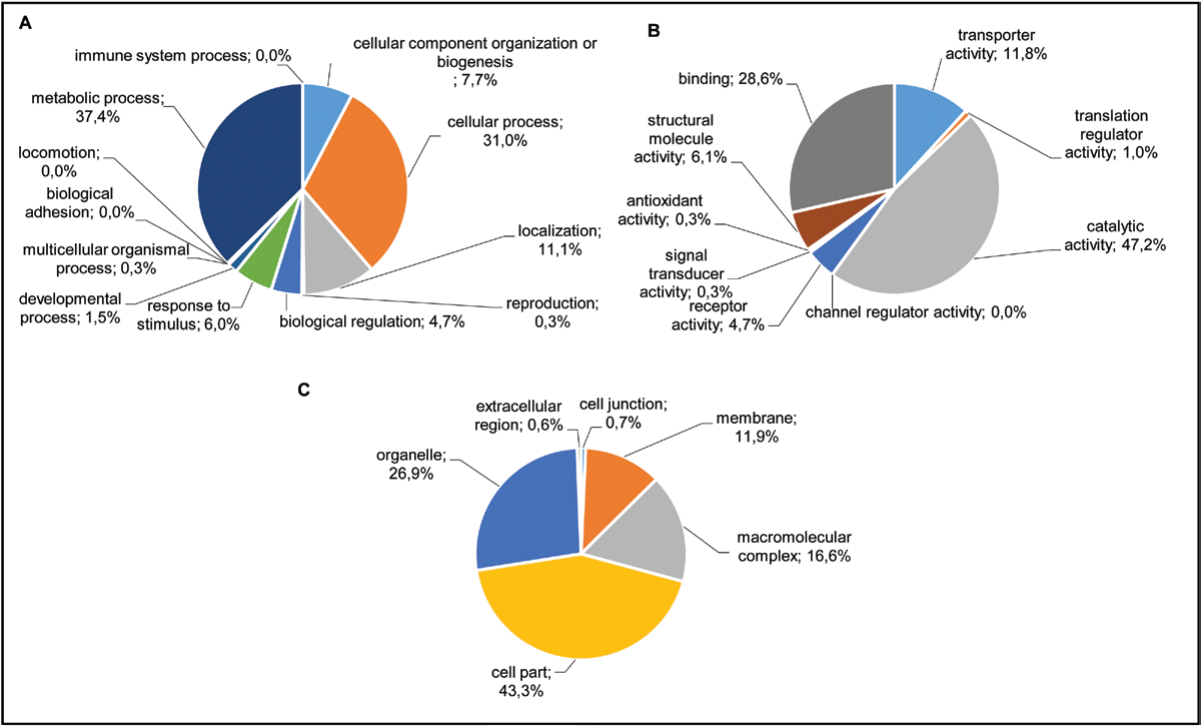
Gene Ontology analyses of all contigs. Pram contigs were assigned to GO slim terms for biological processes (A), molecular functions (B), and cellular components (C) using the PANTHER algorithm (http://www.pantherdb.org) (Mi *et al.*, 2016). Numbers indicate percentages of each GO slim term within main ontologies.

Local alignment using the BLASTx algorithm (cut-off e-value 1 × 10^10^) showed that 51% and 55% of *P. ramosa* contigs/unigenes have homologous sequences in the *A. thaliana* genome (TAIR 10) and in the *S. lycopersicum* (GeneBank 160) genome, respectively (Supplementary Table S3). *Phelipanche ramosa* contigs/unigenes were then classified into 6227 slim GO terms which were regrouped in 30 main terms (Fig. 3A–C, Supplementary Data set S1). Among the terms represented, the biological process category was mainly classified into metabolic process and cellular process, accounting for ~34% and 31% of the transcripts, respectively. The molecular function category was dominated by catalytic activity and binding. Of these, hydrolase activity (31%), transferase activity (29%), oxidoreductase activity (15%), nucleic acid binding (57%), and protein binding (30%) were the most represented. In the cellular component category, 43% of the contigs were classified in cell part, of which 82% account for intracellular and 14% account for plasma membrane localization (Fig. 3A–C; Supplementary Data set S1).

### Identification and characterization of *P. ramosa* genes induced during haustorium formation

**Fig. 4.**
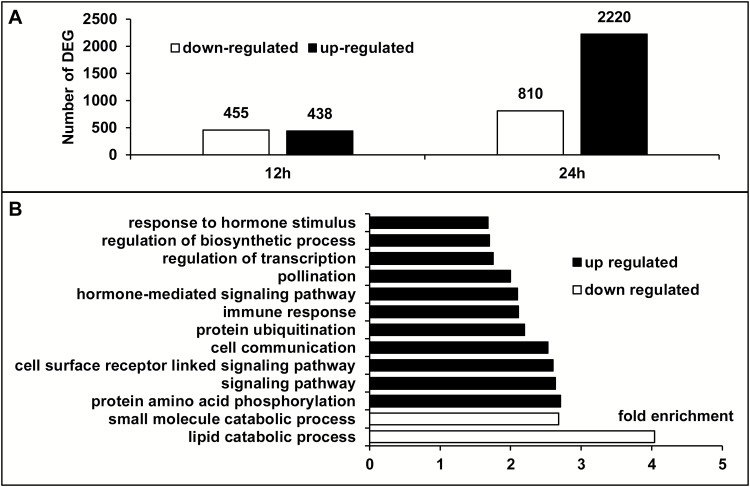
Analysis of differentially expressed genes (DEGs) during haustorial development. (A) Number of DEGs with log_2_ (FC) (fold change between *B. napus* exudate-treated/control *P. ramosa* germinated seeds) ≥1.5 or ≥ –0.75 at 12 h and 24 h. (B) Selected significant GO terms for pooled annotated DEGs. GO term enrichment analysis was carried out on the pooled DEGs using the AGRIGO algorithm. Fisher’s exact test (Hochberg FDR <0.05).

To identify genes involved in haustorium formation and especially genes regulated before the visible onset of EHSs, a microarray based on the *P. ramosa* transcriptome assembly was designed. Germinated seeds were treated with *B. napus* root exudates and gene transcriptional levels were analyzed by microarray hybridization. According to the time course experiment (Fig. 1J), two time points (12 h and 24 h) were chosen for the microarray analysis in order to focus on early transcriptional changes. In these conditions, 893 DEGs displayed a 1.5-fold higher or 0.75-fold lower expression 12 h after treatment, and 3030 were detected after 24 h of treatment (Fig. 4A). RT-qPCR analyses were performed on a set of 21 randomly selected DEGs to validate the microarray results. Expression profiles showed patterns similar to those obtained from microarray analysis, thus demonstrating the reproducibility of microarray data (Supplementary Fig. S2).

DEGs were divided into two pools according to their up- or down-regulation, and the average gene expression values (fold changes against the control sample) was calculated for each pool and displayed in Table 1.

**Table 1.** Average expression values of annotated DEGs as log_2_ (FC) DEGs at 12 h or 24 h were pooled in two clusters according to their over- or under expression status.

Cluster	Sequence number with hit blast *A. thaliana*	Average log _ 2 _ (FC)
Pool up-regulated	974	1.4 ± 0.7
Pool down-regulated	720	–0.8 ± 0.3

Means are values ±SD.

To investigate a functional shift in the gene population, GO term enrichment analyses were carried out on the two pools of DEGs. Among the sequences assigned to the GO term biological process, repressed genes showed an over-representation of GO terms corresponding to lipid catabolic process and small molecule catabolic process. Up-regulated genes were notably enriched for terms corresponding to protein amino acid phosphorylation, signaling pathway, regulation of transcription, and hormone response, which suggests an involvement of hormone perception and signaling in EHS induction (Fig. 4B; Supplementary Data set S2).

### Multiple hormone responses

Among DEGs, 79 genes were related to a broad hormone response (Supplementary Data set S2). Considering this result, the impact of plant hormones on EHS formation was assessed using a set of hormones including auxin [1-naphthaleneacetic acid (NAA)], CK [*c/t*-zeatin (*c/t*Z)], gibberellic acid (GA_3_), abscisic acid (ABA), ethylene [1-aminocyclopropane-1-carboxylic acid (ACC)], jasmonic acid (JA), brassinosteroids (castosterone), and strigolactones (*rac*-GR24) at physiological concentrations ranging from 10^–4^ M to 10^–9^ M. *c/t*Z was the only molecule to induce EHS formation, with ~80% of the radicles harboring these structures at a concentration range of 10^–8^–10^−7^ M (Table 2A). As expected, no induction of EHSs was observed when germinated seeds were treated using molecules previously identified as HIFs in hemiparasitic plants, including DMBQ, syringic acid, vanilic acid, and coniferyl alcohol (Supplementary Table S4A).

**Table 2.** Effect of treatments with phytohormones on EHS induction. (A) *P. ramosa* germinated seeds were treated with an array of phytohormones at concentrations ranging from 10^–4^ M to 10^−10^ M. (B) Inhibiting activities were determined using a co-treatment with either *c/t*Z at optimum concentration (10^–7^ M) and NAA or ABA at concentrations ranging from 10^–4^ M to 10^−10^ M, or root exudates (Ex) and NAA or ABA at concentrations ranging from 10^–4^ M to 10^−10^ M. Results for treatments with *c*/*t*Z or exudates alone are displayed in (A). Exudates were half-diluted in buffer solution (0.5 mM HEPES, 0.05% PPM).

	Compound	EHS induction (%)						
		10^–4^M	10^–5^M	10^–6^M	10^–7^M	10^–8^M	10^–9^M	/
**A**	GR24	NI	NI	NI	NI	NI	NI	
	Cstosterone	NI	NI	NI	NI	NI	NI	
	GA_3_	NI	NI	NI	NI	NI	NI	
	ABA	NI	NI	NI	NI	NI	NI	
	NAA	NI	NI	NI	NI	NI	NI	
	ACC	NI	NI	NI	NI	NI	NI	
	JA	NI	NI	NI	NI	NI	NI	
	*c*/*t*Z	NI	NI	NI	82 ± 4	81 ± 1	NI	
	Exudates	–	–	–	/	/	/	88 ± 2
**B**	*c*/*t*Z (10^–7^ M)+NAA	NI	NI	2 ± 2***	87 ± 2	86 ± 2	87 ± 3	
	*c*/*t*Z (10^–7^ M)+ABA	NI	19 ± 3***	40 ± 2***	85 ± 1	86 ± 1	85 ± 2	
	Ex+NAA	NI	NI	87 ± 2	84 ± 4	88 ± 2	86 ± 3	
	Ex+ BA	36 ± 8***	50 ± 6***	92 ± 2	89 ± 1	95 ± 3	91 ± 2	

NI, non-induced.

Means are values ±SE (*n=*6 wells).

Means annotated with asterisks are significantly different from the highest induction value (*t*-test, ***P<0.001).

These results show that *c/t*Z triggers EHS formation in *P. ramosa* whereas NAA and ABA tended to have an antagonistic action, thus suggesting the involvement of the CK response in this process.

### Direct CK treatments induce EHS at the apex of the *P. ramosa* radicle

**Fig. 5.**
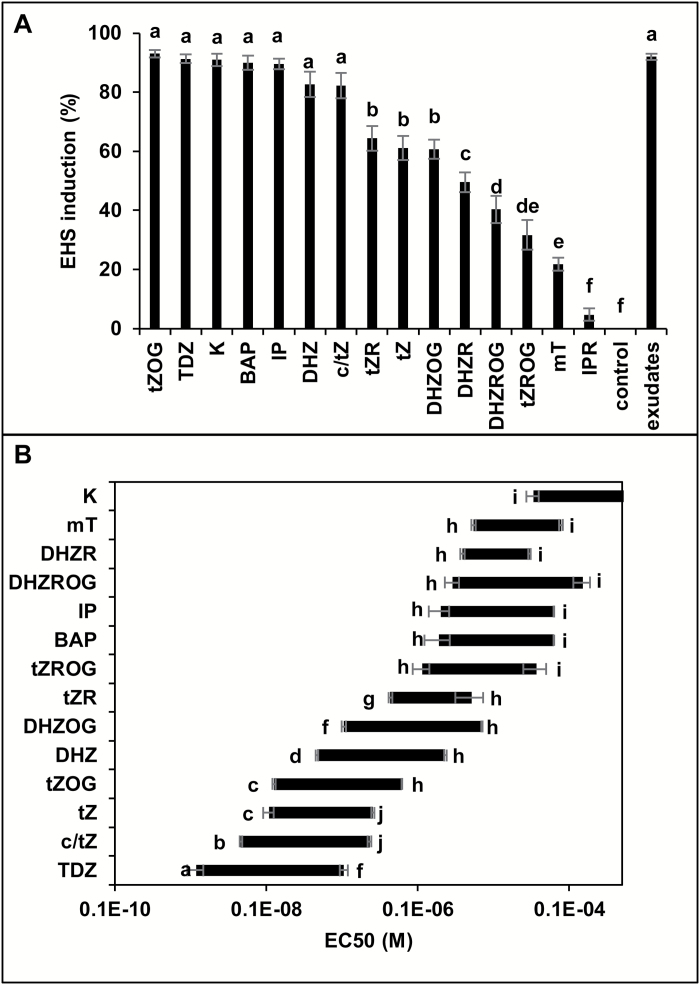
Effect of treatments with cytokinins on EHS induction. (A) Maximum EHS induction at the apex of *P. ramosa* germinated seeds after 72 h of treatment with an optimal concentration of *t*ZOG (10^–7^ M), TDZ (10^–8^ M), K (10^–4^ M), BAP (10^–5^ M), *c*/*t*Z (10^–7^ M), DHZ (10^–7^ M), IP (10^–5^ M), *t*Z (10^–7^ M), DHZOG (10^–6^ M), *t*ZR (10^–6^ M), DHZR (10^–5^ M), DHZROG (10^–5^ M), mT (10^–5^ M), IPR (10^–5^ M), and *t*ZROG (10^–6^ M). Means are values ±SE (*n*=6). Values with the same letter are not significantly different from the control points (ANOVA *P<*0.01). (B) Concentration-dependent stimulation by *t*ZOG, TDZ, K, BAP, *c*/*t*Z, DHZ, IP, *t*Z, DHZOG, *t*ZR DHZR DHZROG, mT, and *t*ZROG with concentrations ranging from 10^–4^ M to 10^−10^ M. EC_50_=half-maximal effective concentration. Means are values ±SE (*n*=6). Values with the same letter are not significantly different from the control points (ANOVA *P<*0.01).

To confirm the role of CKs in EHS development, germinated seeds were treated independently with a set of 15 adenine-type CKs and phenylurea-type CKs known for their different structure–activity relationship (SAR) (Holub *et al.*, 1998; Spíchal, 2012). Adenine-type CKs include biologically active CKs [isopentenyladenine (IP), zeatin (Z), and dehydrozeatin (DHZ)], glycosylated and ribosylated CKs known to have reduced biological activities [zeatin *O*-glucoside (ZOG), DHZ *O*-glucoside (DHZOG), IP riboside (IPR), Z riboside (ZR), DHZ riboside (DHZR), DHZ riboside-*O*-glucoside (DHZROG), and *t*Z riboside-*O*-glucoside (*t*ZROG)], and 6-benzyl, 6-furfuryl, and 6-hydrobenzyl CKs [6-benzylaminopurine (BAP), kinetin (K), and meta-tomalin (mT)]. The highly biologically active phenylurea-type CK thidiazuron (TDZ) was also included (Supplementary Table S1). For each CK, the maximum haustorium induction activity (Fig. 5A) and the dose–response activity (Fig. 5B) were determined for concentrations ranging between 10^−10^ M and 10^–3^ M. Interestingly, as shown in Fig. 5B, each CK displayed two EC_50_ values, meaning that the efficiency of the compound can be determined with the concentration value but also with the active concentration range. In these conditions, TDZ was the most efficient compound with a maximum activity of 91 ± 1% (Fig. 5A) and active concentrations ranging from 1.2 × 10^−9^±2.8 × 10^−10^ M to 10^–7^±1.3 × 10^−8^ M (Fig. 5B). The most active group of CKs contains the isoprenoid CKs *t*ZOG, *c/t*Z, DHZ, IP, and the aromatic CKs K and BAP, inducing >80% of EHSs. A second group was formed with *t*Z, DHZOG, and *t*ZR, with an induction activity found between 60% and 70%. DHZR induced 50 ± 8% of EHSs and, below this value, a last group with the least active CKs (DHZROG and *t*ZROG) was found (Fig. 5A). Taken together, these results suggest a strong role for CKs in EHS development.

However, CKs with glycosylated or aromatic substituents display reduced efficiency. Indeed, it was found to be reduced 2–5 times when a glucose substituent was added (*t*Z>*t*ZOG; DHZ>DHZOG; DHZR>DHZROG) and 20–70 times when a ribose substituent was added (tZ>tZR; DHZ>DHZR; DHZOG>DHZROG). The addition of furfuryl or hydroxybenzyl substituents also decreased the overall efficiency (Fig. 5B).

### Cytokinin receptor inhibitor decreases EHS formation and parasite aggressiveness

**Fig. 6.**
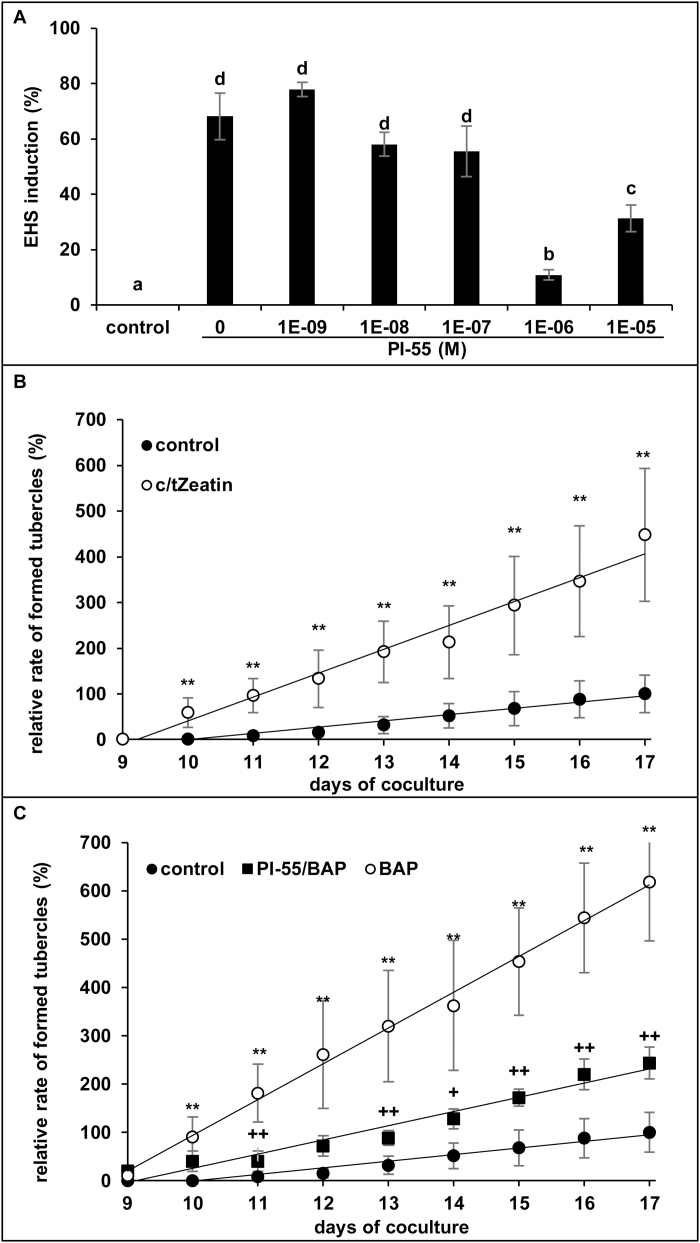
Induction of EHSs and parasite aggressiveness are controlled by cytokinins. (A) *P. ramosa* germinated seeds were co-treated with BAP (10^−5^ M) and PI-55 at concentrations ranging from 10^–6^ M to 10^–9^ M. The control condition corresponds to a treatment with BAP (10^–5^ M) alone. Means are values ±SE (*n*=6). Means annotated with different letters are significantly different from the control point (*t*-test, **P<0.001). (B) A 20 mg aliquot of *P. ramosa* germinated seeds was incubated for 48 h in *c*/*t*Z (10^–7^ M) or control solution before placing them in contact with *B*. *napus* roots. Five plantlets per plate and five plates per condition were used. The number of developing tubercles was assessed using the phloem-mobile dye 6-carboxyfluorescein and the results were expressed as a percentage of the maximum number of tubercles observed in control conditions. The control condition corresponds to a treatment with the buffer solution alone (0.5 mM HEPES, 0.05% PPM). Means are values ±SE (*n*=5). Means annotated with asterisks are significantly different from the control points (*t*-test, *P<0.05 and **P<0.01). (C) A 20 mg aliquot of *P. ramosa* germinated seeds was incubated for 48 h in BAP (10^–5^ M), BAP (10^–5^ M)/PI-55 (10^–6^ M), or control solution before placing the seeds in contact with *B*. *napus* roots. Five plantlets per plate and five plates per condition were used. The control condition corresponds to a treatment with the buffer solution alone (0.5 mM HEPES, 0.05% PPM). Means are values ±SE (*n*=5). Means annotated with astericks are significantly different from the control points (*t*-test, **P<0.01). Means annotated with plus signs are significantly different from the BAP alone points (*t*-test, ^+^*P<*0.05 and ^++^*P<*0.01, respectively).

To determine if CK perception is crucial for EHS formation in *P. ramosa*, PI-55, a specific inhibitor of BAP perception, was used. PI-55 is structurally related to BAP and was shown to inhibit competitively BAP binding on Arabidopsis-specific receptors CRE1/AHK4 and AHK3 (Spíchal *et al.*, 2009). Since the *P. ramosa* transcriptome also contains CRE1/AHK4 homolog sequence (Pram_00096), germinated seeds were treated with 10^–5^ M BAP alone or concomitantly with PI-55 (concentrations ranging from 10^–5^ M to 10^–9^ M) for 72 h, and EHS induction was monitored. BAP alone induced 68 ± 8% of EHSs. A maximum reduction of 83.8% was observed at 10^–6^ M PI-55 in the percentage of EHS induction (Fig. 6A).

In order to determine the involvement of CKs in the aggressiveness of *P. ramosa*, a co-cultivation assay was used. Germinated seeds were treated or not with either 10^–7^ M *c/t*Z or 10^–5^ M BAP or with 10^–5^ M BAP together with 10^–6^ M PI-55 for 48 h before placing them in contact with roots of *B. napus* plantlets. The number of tubercles in *c/t*Z- and BAP-treated conditions was already significantly higher (59 ± 32% and 91 ± 42%, respectively) than in untreated conditions (0%) from the 10th day on, and reached 448 ± 145% and 619 ± 123%, respectively, at the end of the experiment (Fig. 6B, C). During co-treatment with BAP/PI-55, a 56% reduction in the number of tubercles was observed at 10 d and a 60.5% reduction was observed at 17 d (Fig. 6C).

These results demonstrate that pre-inducing the development of EHS with CKs clearly increased the aggressiveness of the parasite in a similar way to that observed with *B. napus* root exudates, and that the inhibition of CK perception decreased both the number of EHSs and the subsequent aggressiveness of the parasite.

### Root exudates induce a cytokinin response pattern

**Fig. 7.**
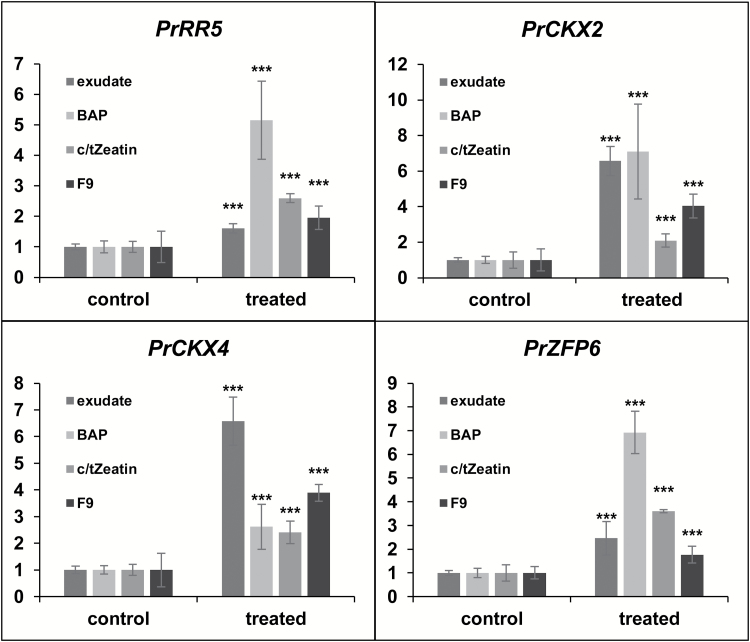
*Brassica napus* root exudates trigger the expression of cytokinin-responsive genes. (A–C) Expression levels of cytokinin-responsive genes (*PrRR5*, *PrCKX2*, *PrCKX4*, and *PrZFP6*) in *P. ramosa* germinated seeds treated for 24 h with either *B. napus* root exudates, BAP (10^–5^ M), *c/t*Z (10^–7^ M), or purified fraction 9 measured by RT-qPCR. The values correspond to the fold change compared with the control treated germinated seeds. The control condition corresponds to a treatment with buffer solution (0.5 mM HEPES, 0.05% PPM) supplemented with the corresponding solvent. Exudates and fraction 9 were half-diluted and 130-fold diluted in buffer solution (0.5 mM HEPES, 0.05% PPM), respectively. Two biological replicates were performed, each in three technical replicates. Means are values ±SD (*n*=6). Means annotated with asterisks are significantly different from the control points (*t*-test, ***P<0.001).

To determine if the formation of EHSs induced by *B. napus* root exudates was accompanied by a similar gene response to that observed in response to CKs, *P. ramosa* germinated seeds were treated with exudates, 10^–7^ M *c/t*Z, 10^–5^ M BAP, or control buffer for 24 h. Total RNA was extracted and qRT-PCR experiments were carried out on a set of four genes (*PrRR5*, *PrCKX2*, *PrCKX4*, and *PrZFP6*), which were found to be differentially expressed in the present microarray analysis (Supplementary Data set S2) and which are described as CK marker genes (Bhargava *et al.*, 2013). As observed in Fig. 7, an accumulation of transcripts in response to either exudates or CK treatments was observed in the case of all marker genes, whereas no induction was observed in control conditions, which suggests that *B. napus* root exudates trigger a CK-like transcriptional response. Moreover, concomitant treatments with root exudates or *c/t*Z at optimum concentration (10^–7^ M) together with known antagonists of CK (NAA or ABA) decreased the number of EHSs at the apex of *P. ramosa* radicles (Table 2B). Indeed, induction triggered by *c*/*t*Z at 10^–7^ M or root exudates was completely abolished at 10^–6^ M and 10^–5^ M NAA, respectively. An ABA co-treatment at 10^–6^ M and 10^–5^ M decreased the induction triggered by *c*/*t*Z at 10^–7^ M or root exudates of ~50% of ABA.

### Characterization of HIFs released by *Brassica napus* roots

**Fig. 8.**
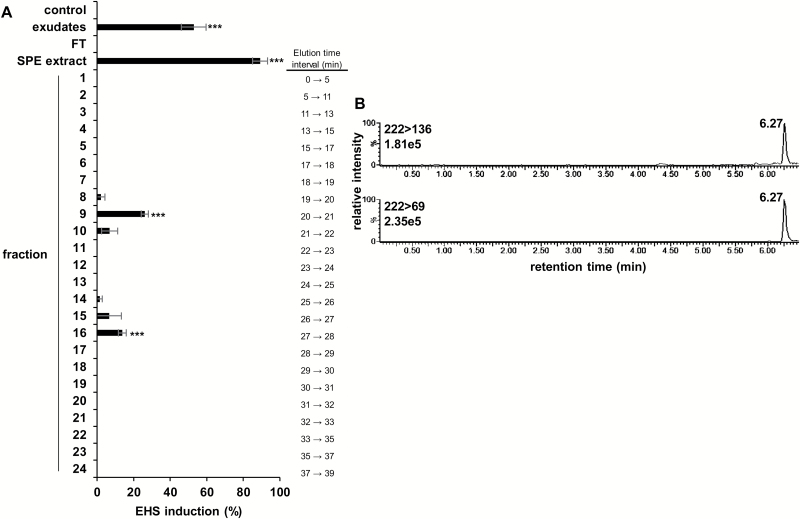
Fractionation of *B. napus* root exudates. (A) Distribution of EHS induction activity after RP-HPLC fractionation of SPE concentrated *B. napus* root exudates. Half-diluted exudates and SPE extracts (1000-fold diluted) were used as positive controls, Coïc medium was used as negative control. HPLC-purified fractions were 100-fold diluted. All dilutions were performed in buffer solution (0.5 mM HEPES, 0.05% PPM). Means are values ±SE (*n*=3). Means annotated with asterisks are significantly different from the negative control (*t*-test, ***P<0.001). (B). UPLC-ESI(+)-MS/MS analysis of fraction 9 detected in the MRN channel of DHZ.

*Brassica napus* plants were grown hydroponically for 5 weeks and root exudates from weeks 3, 4, and 5 were collected and assayed for EHS induction. Active crude exudates were pooled, extracted by SPE, and separated by a RP-HPLC in 24 fractions. EHS induction was assessed throughout the purification process. The activity of *B. napus* root exudates was efficiently concentrated in the SPE extract (Fig. 8A). Regarding the different RP-HPLC fractions, EHS induction activity was significantly associated with only fractions 9 and 16 which eluted at 20–21 min and 27–28 min, respectively (Fig. 8A). The retention times of each fraction were compared with those of known CKs and with compounds previously tested for haustorium induction activity in hemiparasites (Kim *et al.*, 1998; Albrecht *et al.*, 1999). The results showed that fraction 16 (27–28 min) co-eluted with isorhamnetin which had no haustorium induction activity on *P. ramosa* (Supplementary Tables S4A, S5B), and interestingly fraction 9 co-eluted with different CK standards displaying EHS induction, including *c*/*t*Z, *t*Z, *t*ZR, *t*ZROG, DHZ, DHZR, and DHZROG (Supplementary Table S5A; Fig. 5A, B).

All fractions were further examined for the presence of 12 known CKs by using UPLC-ESI(+)-/MS/MS. No tested CK standard was detected in any fractions. Nevertheless, two transitions of *m/z* 222>136 and 222>69 normally associated with DHZ were detected specifically in fraction 9 at a retention time of 6.27 min (Fig. 8B; Supplementary Table S6). These results show that the active fraction 9 contains a CK which shares a DHZ structure. Moreover, as observed in response to crude root exudates, fraction 9 also showed a CK-inducing potential at the gene expression level since all four tested marker genes were induced in response to this fraction (Fig. 7).

## Discussion

The molecular determinism of the key development step of haustorium formation is largely unknown in root holoparasitic plants (Joel and Losner-Goshen, 1994). In the present study, we demonstrated that radicles of *P. ramosa* germinated seeds treated with uninfected *B. napus* root exudates displayed a growth arrest, a swollen apex, and epidermal cell outgrowth corresponding to EHS development previously described in either hemiparasitic or holoparasitic plants (Baird and Riopel, 1983; Joel and Losner-Goshen, 1994; Bandaranayake and Yoder, 2013; Cui *et al.*, 2016). These results show that *B. napus* roots constitutively produce active molecules which trigger durable changes in cell expansion, cell division, and cell differentiation at the apex of *P. ramosa* radicles.

Haustorium formation has been studied for decades in either holoparasites or facultative and obligate hemiparasitic plants and, whereas EHS formation has been shown to be an inducible process in the latter, the role of these structures in the overall aggressiveness of the parasite has long been uncertain and linked to the presence of attachment substances at the surface of haustorial hairs or papillae (Baird and Riopel, 1983; Joel and Losner-Goshen, 1994). The present study highlights the role of EHS development in *P. ramosa* aggressiveness. Indeed, a strong increase in the number of broomrape attachments (tubercles) was observed when germinated seeds were pre-treated with *B. napus* root exudates to induce EHSs before placing them in contact with *B. napus* roots (Fig. 2). Similarly, a recent study in *P. japonicum* showed that plants displaying the *haustorial hair defective* (*hhd*) mutation fail to form haustorial hairs upon DMBQ treatment and form fewer functional haustoria when put in contact with host roots (Cui *et al.*, 2016).

The transcriptome assembly obtained in this work yielded 53 511 contigs, 51% and 55% of which have a BLASTx hit with the Arabidopsis TAIR 10 database and the *S. lycopersicum* (GeneBank 160) genome, respectively. Although this value is weaker than the one found in a recent study in *P. japonicum* (60.23%) with 57 939 unigenes (Ishida *et al.*, 2016), our *de novo* assembled transcriptome still provides a reasonable coverage of *P. ramosa* genes. The *P. ramosa* microarray analysis focused on time points surrounding EHS formation in response to *B. napus* root exudates. GO term enrichment analysis of the overall DEGs did not reveal any over- representation of GO terms linked to reactive oxygen species (ROS) generation or response but an under-representation of the GO term GO:0016491 oxidoreductase activity. This finding differs from the over-representation of this GO term observed in previous transcriptomic analyses focusing on haustorium development in hemiparasitic species in response to DMBQ treatment (Yang *et al.*, 2015; Ishida *et al.*, 2016). Indeed, although ROS-related genes have been previously proposed to play an important role in the signaling leading to haustorium development in these species (Matvienko *et al.*, 2001*b*; Bandaranayake *et al.*, 2010; Ngo *et al.*, 2013; Yang *et al.*, 2015; Ishida *et al.*, 2016), ROS-related compounds such as DMBQ have no effect on haustorium formation in *P. ramosa*. The GO term pollination (GO:0009856) was over-represented in *P. ramosa* germinated seeds in response to root exudates, which might be surprising but it has previously been proposed that haustorial growth may be related to pollen tube growth (Thorogood and Hiscock, 2010; Pérez-de-Luque, 2013), and genes linked to this process were also detected during haustorium formation in hemiparasitic plants (Yang *et al.*, 2015). The GO analysis also revealed an increase inthe expression of genes related to signaling pathway, hormone-mediated signaling pathway, or regulation of transcription. This shift in the GO terms underlines the involvement of hormone signaling, which was previously described during haustorium development (Tomilov *et al.*, 2005; Ishida *et al.*, 2016). Indeed, auxin induces the formation of lateral haustoria in *T. versicolor* and *P. japonicum*. In contrast, it inhibits haustorium formation in the obligate hemiparasite *S. asiatica* which, like *P. ramosa*, forms terminal haustoria (Keyes *et al.*, 2000; O’Malley and Lynn, 2000; Tomilov *et al.*, 2005; Ishida *et al.*, 2016). The present study confirms those results since auxin did not show any EHS induction on *P. ramosa* germinated seeds and had an inhibitory effect when applied together with root exudates.

Among the different plant hormones tested in this study, only *c*/*t*Z triggered EHS formation with a maximum activity similar to *B. napus* root exudate treatment. Although the synthetic CK kinetin has already been shown to act in a similar way to DMBQ by inducing EHS formation in *S. asiatica* (Keyes *et al.*, 2000; O’Malley and Lynn, 2000), the swollen apex induced by this hormone was described as smaller than the one induced by DMBQ treatment whereas haustorial hair growth was exaggerated, giving rise to a somewhat distorted haustorium with no demonstrated functionality. The results presented here show that in contrast to DMBQ which has no effect, CKs trigger EHS formation in *P. ramosa* and increase the parasite aggressiveness. Moreover, when an array of CKs was tested, the SAR observed during EHS induction matched the one previously described in other development processes such as callus growth (Matsubara, 1980). The CK signal is mediated by the histidine kinase receptors CRE1/AHK4, AHK3, and AHK2 in *A. thaliana* (Inoue *et al.*, 2001; Suzuki *et al.*, 2001; Ueguchi *et al.*, 2001; Yamada *et al.*, 2001; Nishimura *et al.*, 2004), which act as a two-component signaling pathway (Hwang *et al.*, 2002). PI-55 is a purine derivative which specifically competitively inhibits the binding of BAP to CRE1/AHK4 in *A. thaliana* and thus disrupts this two-component signaling pathway. However, this molecule also displays a partial agonist activity on CK perception (Spíchal *et al.*, 2009). In the present study, the co-treatment of *P. ramosa* germinated seeds with PI-55 together with BAP yielded a massive reduction in the number of the observed EHSs but without completely abolishing their formation. Pharmacological approaches using PI-55 usually fail to be 100% successful. PI-55 effectively blocks the activity of CRE1/AHK4 CK receptor in Arabidopsis, but at high concentrations it has its own weak CK activity, most probably through another receptor (AHK3) (Spíchal *et al.*, 2009). This could explain the observed ‘mixed’ behavior of PI-55 at high concentrations (10^−6^ M and 10^−5^ M), which can cause an incomplete blocking of EHS development, especially when CK activity promotes it. PI-55 treatment also reduced the overall aggressiveness of *P. ramosa* when applied with BAP in comparison with BAP alone, suggesting that the signaling pathway leading to EHS formation involves histidine kinase receptors homologous to CRE1/AHK4 and AHK3. A transcript accumulation analysis of a set of known CK-responsive genes conducted during EHS induction in *P. ramosa* with either *B. napus* root exudates or CKs (*c*/*t*Z and BAP) revealed similar gene expression patterns. Interestingly, bio-guided RP-HPLC fractionation of *B. napus* root exudates revealed that two fractions (9 and 16) displayed high EHS induction activities and fraction 9 co-eluted with *c*/*t*Z, *t*Z, *t*ZR, *t*ZROG, DHZ, DHZR, and DHZROG standards. A subsequent UPLC-ESI(+)-/MS/MS analysis revealed that only the active fraction 9 contained a molecule displaying the transition *m*/*z* 222>136 and 222>69, which are normally associated with DHZ. This finding is also concordant with the CK-inducing potential of this fraction here demonstrated at the gene expression level. Although a further determination of the exact structure of this CK is required as well as the identification of the active compound contained in fraction 16, these results suggest that *B. napus* root exudates contain CKs. Such a finding is in accordance with previous results since the presence of plant-derived CKs has already been mentioned in the rhizosphere of tomato and rice (Davey and van Staden, 1976; Soejima *et al.*, 1992; Yang *et al.*, 2002).

CKs are known to control *A. thaliana* root growth and shape through multiple nodes of interaction with auxin (Schaller *et al.*, 2015). In this complex interplay, CKs tend to inhibit root growth by reducing the size of the root apical meristem and to promote cell differentiation (Dello Ioio *et al.*, 2007) through the involvement of the Aux/IAA protein SHY2 (Dello Ioio *et al.*, 2008; Moubayidin *et al.*, 2010). This demonstrated role of CKs is consistent with the phenotypical changes such as root growth arrest and differentiation of papillae from epidermal cells that occur upon *B. napus* root exudate or direct CK treatments at the root apex of *P. ramosa* germinated seeds. Ishida *et al.* (2016) have shown that auxin levels increased during haustorium induction and that the overexpression of *YUC3*, an auxin biosynthesis gene, was sufficient to induce EHS formation in the hemiparasitic species *P. japonicum*. A direct auxin treatment does not induce EHS formation in obligate root parasitic plants such as *P. ramosa* and *S. hermontica* that form terminal haustoria (Keyes *et al.*, 2000; O’Malley and Lynn, 2000; this study). However, it is effective in facultative hemiparasitic plants that form lateral haustoria (Tomilov *et al.*, 2005; Ishida *et al.*, 2016). Interestingly, CKs have been shown to be effective in both obligate and facultative parasitic plants. Although this suggests that CKs and auxin may interact to shape lateral haustorium formation in facultative parasitic plants and CKs alone may govern the establishment of the terminal haustorium in obligate root parasitic plants, more detailed studies are required on the involvement of such an interplay in this specific root development.

The results presented here demonstrate that EHS formation in *P. ramosa* is an inducible process under the control of host root exudate compounds, notably of an as yet unidentified CK contained in *B. napus* root exudates. This sheds new light on the establishment of the haustorium in parasitic plants by putting an emphasis on the involvement of host-exudated CKs as HIFs.

## Supplementary data

Supplementary data are available at *JXB* online.

Table S1. Stock solution used.

Table S2. List of primers used for RT-qPCR analysis.

Table S3. Summary of the *de novo* assembly of the *P. ramosa* transcriptome.

Table S4. Effect of treatments with known HIFs and solvent effect on haustorium formation in *P. ramosa*.

Table S5. Chromatographic behavior of standard compounds during RP-HPLC fractionation.

Table S6. Chromatographic behavior of cytokinin standard compounds during UPLC-ESI(+)-MS/MS analysis.

Fig. S1. Dose–response effect of exudate dilution.

Fig. S2. RT-qPCR validation of the expression profiles of selected genes.

Data set S1. GO term enrichment analysis on *de novo* assembly of the *P. ramosa* transcriptome.

Data set S2. GO term enrichment analysis on the DEG set.

Data deposition. Transcriptome sequence data have been deposited in the Parasitic Plant Genome Project (Westwood *et al.*, 2012; Yang *et al.*, 2015; http://ppgp.huck.psu.edu/). Microarray data have been deposited in the Gene Expression Omnibus under the accession numbers GPL23512 and GSE99377.

## Supplementary Material

supplementary_Tables_and_Figures

supplementary_dataset_S1

supplementary_dataset_S2

## Abbreviation:

CK: cytokinin
DEG: differentially expressed gene
EHS: early haustorial structure
GO: Gene Ontology
HIF: haustorium-inducing factor.
