# Supplementary material for: Haustorium initiation in the obligate parasitic plant Phelipanche ramosa involves a host-exudated cytokinin signal
Source: J Exp Bot. 2017 Oct 21;68(20):5539–52. doi: 10.1093/jxb/erx359 (PMC5853424; doi:10.1093/jxb/erx359)
Supplement: supplementary_Tables_and_Figures [file erx359_suppl_supplementary_tables_and_figures.pdf]

Supplementary data

Table S1: Stock solutions used

Each compound was prepared as stock solution in the corresponding solvant and stored at -20°C. *c/t*Z, *t*Z, *t*ZR, *t*ZOG, *t*ZROG, DHZ, DHZR, DHZOG, DHZROG, IPR and *m*T were purchased from OlChemIm (Czech Republic). IP, K, BAP, TDZ, GA3, ABA, NAA, ACC, JA, syringic acid, vanilic acid, DMBQ, coniferyl alcohol, p-coumaric acid, vanillin, and quercetin were purchased from Sigma Aldrich. Isorhamnetine and kaempferole were purchased from Extrasynthèse (France). *rac*-GR24 was provided by Dr. Binne Zwanenburg. PI-55 was provided by Dr. Lukáš Spíchal.

**compound**		**stock concentration**	**solvant solubilisation**
**name**	**abbreviation**		
cis/*trans* zeatin	*c/t*Z	10 µM	ACN 50%
trans zeatin	*t*Z	10 µM	ACN 50%
trans zeatin riboside	*t*ZR	10 µM	ACN 50%
trans zeatin O-glucoside	*t*ZOG	10 µM	ACN 50%
trans zeatin riboside O-glucoside	*t*ZROG	10 µM	ACN 50%
dihydrozeatin	DHZ	10 µM	ACN 50%
dihydrozeatin riboside	DHZR	10 µM	ACN 50%
dihydrozeatin O-glucoside	DHZOG	10 µM	ACN 50%
dihydrozeatin riboside O-glucoside	DHZROG	10 µM	ACN 50%
isopentenyl adenine	IP	10 µM	ACN 50%
isopentenyl adenosine	IPR	10 µM	ACN 50%
meta-topolin	*m*T	10 µM	ACN 50%
kinetin	K	10 µM	ACN 50%
6-benzylaminopurine	BAP	10 µM	ACN 50%
thidiazuron	TDZ	10 µM	ACN 50%
racemic GR24	*rac*-GR24	10 µM	ACN 50%
giberrelin 3	GA3	10 µM	ACN 50%
abscisic acid	ABA	10 µM	ACN 50%
1-naphthaleneacetic acid	NAA	10 µM	ACN 50%
1-aminocyclopropane-1-carboxylic acid	ACC	10 µM	ACN 50%
castosterone	/	10 µM	DMSO 100%
jasmonic acid	JA	500 µM	DMSO 100%
6-(2-hydroxy-3-methylbenzylamino)purine	PI-55	50 µM	DMSO 100%
syringic acid	/	10 µM	DMSO 100%
vanilic acid	/	10 µM	DMSO 100%
2,6-Dimethoxy-1,4-benzoquinone	DMBQ	10 µM	DMSO 100%
coniferyl alcohol	/	10 µM	DMSO 100%
p-coumaric acid	/	10 µM	DMSO 100%
vanillin	/	10 µM	DMSO 100%
quercetin	/	10 µM	DMSO 100%
isorhamnetin	/	10 µM	DMSO 100%
kaemferol	/	10 µM	DMSO 100%

Table S2: List of primers used for RT-qPCR analysis

Primers were ordered as salt-free-purified from Eurofins Genomics Company (Ebersberg, Germany)

seq_id	primer_id	sequence
Pram_21119	Pram_21119-F	GCAGGAGGTTCCCTTGGAA
	Pram_21119-R	GGTTGCTCCCAGGACTTGAG
Pram_46434	Pram_46434-F	AACGATGCAGCAGACATGGA
	Pram_46434-R	GCAGGAATAGCAGAGTTGTGTTTG
Pram_43760	Pram_43760-F	ACGCACACAAGAAGGAGAGACA
	Pram_43760-R	TTCGGCTAGCCTGAATTTGC
Pram_35892	Pram_35892-F	AGGGTCGGTTCGAGAAAAGC
	Pram_35892-R	CCCCATTGCCACGTGAAG
Pram_16837 (PrRR5)	Pram_16837-F	TCCTTTATCGGTGTCGGTATCC
	Pram_16837-R	GGCGGCAGCACCATAGG
Pram_42581 (PrCKX4)	Pram_42581-F	AAATGCATGTCTGCGGACAA
	Pram_42581-R	TTCTATAATGGCATTGGCATCGT
Pram_42809 (PrCKX2)	Pram_42809-F	CGTGTTTGCCAAAAGACTGACA
	Pram_42809-R	TCTTTCGTGGTTTTCCAATTAGG
Pram_08523	Pram_08523-F	GGGCTGCTCAATACTTGTACGA
	Pram_08523-R	CTTCAAAGTCGGGTCGATCAA
Pram_43858	Pram_43858-F	ACGGCACTTTGCTGGTCAATA
	Pram_43858-R	TGCCCGGGCCGTAATT
Pram_15221	Pram_15221-F	GGGTGGCATTAGGTTCACCAT
	Pram_15221-R	CCGACGTTGGTGACGAGTACTA
Pram_11045	Pram_11045-F	CCCTCCGGCGGGTAATT
	Pram_11045-R	GGAAGCTCAATGTCCGTGAAA
Pram_22883	Pram_22883-F	GTCGTGCTCCTTGAACTCATCA
	Pram_22883-R	CGCCTTTACTAGCCGTCTTGA
Pram_45828	Pram_45828-F	CGTGGGCAAGAGCAAACC
	Pram_45828-R	CCAGCCGCAGTGGAAATT
Pram_40182	Pram_40182-F	GAATGCAAGCTTCTTCCGTAGTCT
	Pram_40182-R	GTGGGCAAAGGGTTTTCGA
Pram_41284	Pram_41284-F	GCTATGAATGATAGTGCTGAGGTTCA
	Pram_41284-R	TCAAGAAGCAGCTCCATCCAT
Pram_46808	Pram_46808-F	CCGAACGACGGACAATACGT
	Pram_46808-R	TTCCCGGAGATAAAAAATTCGA
Pram_05576	Pram_05576-F	GGCAGCAGCAATTTTTGAGAAC
	Pram_05576-R	CCGGGCAACCCCAATC
Pram_10105	Pram_10105-F	CCTGTCGGAAGAGCAGACTGTT
	Pram_10105-R	AATCCAGAACGCTCCATTCCT
Pram_00316	Pram_00316-F	TGCCGAAATTCTCAAAGATCGT
	Pram_00316-R	GCACTCGCTACATCATTGCAA
Pram_08710	Pram_08710-F	GGTCGACACGTCTCGTACGA
	Pram_08710-R	GTCCGACGAAGCGAAAACTT
Pram_00348	Pram_00348-F	GCTGCCCGCTGTCAAGA
	Pram_00348-R	GGCGAGTCACCAAGTTGAACA
Pram_00012 (18s)	Pram_00012-F	CCAACTGCGCGCTAACCTA
	Pram_00012-R	CCGTCCTGCTGTCTTAATCGA

Table S3: Summary of the *de novo* assembly of the *P. ramosa* transcriptome. BlastX with threshold e-value less than 10^{-10}.

	**values**
reads	1,251,288
number of bases	50,967,042
number of contigs	53,511
maximum length (bp)	7,872
minimum length (bp)	100
average length (bp)	952
N50 length (bp)	1,071
blast hits to *A. thaliana*	27,448 (51%)
blast hits to S. *lycopersicum*	29,380 (55%)
UCO hits	351 (98%)
APVO hits	797 (82%)

Table S4: Effect of treatments with known HIF and solvent effect on haustorium formation in *P. ramosa*. **(A)** *P. ramosa* germinated seeds were treated for 72h with HIF with concentration ranging from 10^{-4} M to 10^{-10} M. (B) *P. ramosa* germinated seeds were treated either with solvents (DMSO and ACN) in concentration ranging from 1% to 0.00001% or in co-treatment with half diluted *B. napus* root exudates. Exudates were half diluted in buffer solution (HEPES 0.5 mM, PPM 0.05 %). Means are values ± SE (n = 6). NI (non induced).

A	**compound**	**early haustoria induction (%)**						
		**10^{-4} M**	**10^{-5} M**	**10^{-6} M**	**10^{-7} M**	**10^{-8} M**	**10^{-9} M**	**/**
	DMBQ	NI	NI	NI	NI	NI	NI	
	syringic acid	NI	NI	NI	NI	NI	NI	
	vanilic acid	NI	NI	NI	NI	NI	NI	
	vanillin	NI	NI	NI	NI	NI	NI	
	p-coumaric acid	NI	NI	NI	NI	NI	NI	
	coniferyl alcohol	NI	NI	NI	NI	NI	NI	
	quercetin	NI	NI	NI	NI	NI	NI	
	isorhamnetin	NI	NI	NI	NI	NI	NI	
	kaempferol	NI	NI	NI	NI	NI	NI	
	exudates	/	/	/	/	/	/	88 ± 2

B	**compound**	**early haustoria induction (%)**						
		**1%**	**0.1%**	**0.01%**	**0.001%**	**0.0001%**	**0.00001%**	**/**
	exudates	/	/	/	/	/	/	88 ± 2
	DMSO	NI	NI	NI	NI	NI	NI	
	DMSO + Ex	93% ± 2	93% ± 2	91% ± 1	95% ± 1	93% ± 2	92% ± 1	
	ACN	NI	NI	NI	NI	NI	NI	
	ACN + Ex	80 ± 3	86 ± 4	85 ± 4	81 ± 4	82 ± 4	86 ± 4	

Table S5: Chromatographic behavior of standard compounds during reversed phase high-performance liquid chromatography (RP-HPLC) fractionation.

Cytokinin standards **(A)** and phenolic HIF **(B)** were prepared as a 10^{-5} M solution in ACN 50 %, 0.1 % acetic acid. Ninety microliters were loaded onto the column.

A

**standard identity**	**retention time**	**fraction**
trans zeatin O-glucoside	19.55	8
dihydrozeatin O-glucoside	19.79	8
trans zeatin	20.13	9
dihydrozeatin	20.19	9
trans zeatin riboside O-glucoside	20.37	9
trans zeatin riboside	20.41/20.90	9
cis zeatin	20.42	9
dihydrozeatin riboside O-glucoside	20.46	9
dihydrozeatin riboside	20.60/20.95	9
meta-topolin	21.54	10
kinetin	22.34	11
isopentenyl adenine	22.90	11
isopentenyl adenosine	23.48	12

B

**standard identity**	**retention time**	**fraction**
syringic acid	22.51	11
vanilic acid	22.59	11
DMBQ	23.44	12
coniferyl alcohol	23.49	12
p-coumaric acid	23.55	12
vanillin	24.34	13
quercetin	25.91	14
isorhamnetin	27.90	16
kaemferol	29.08	18

Table S6: Chromatographic behavior of cytokinin standards during UPLC-ESI(+)-MS/MS analysis.

standard	retention time	precursor		products	
tZ	5.71	220	148	136	119
tZOG	5.87	382	220	202	136
DHZ	5.98	222	148	136	69
cZ	6.11	220	148	136	119
DHZOG	6.28	384	222	204	136
tZOGR	6.96	514	382	220	202
mT	7.02	242	136	107	77
DHZOGR	7.15	516	384	222	204
tZR	7.13	352	220	202	148
DHZR	7.19	354	222	148	136
IP	8.30	204	148	136	119
IPR	9.20	336	204	148	136

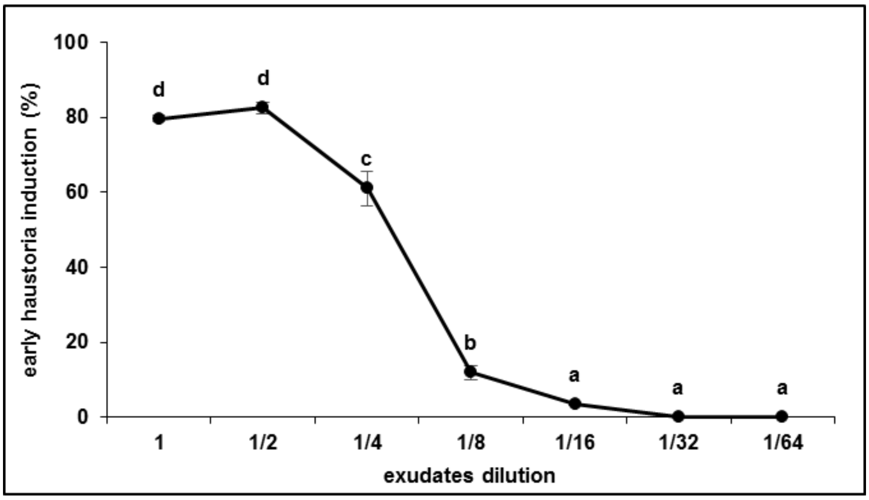

Fig. S1: Dose-response effect of exudate dilution.

P. ramosa germinated seeds were treated with decreasing concentrations of *B. napus* root exudates for 72hrs. Exudates were diluted in buffer solution (HEPES 0.5 mM, PPM 0.05 %). Means are values ± SE (n = 6). Values with the same letter are not significantly different from the control points (Analysis of variance [ANOVA] p <0.001).

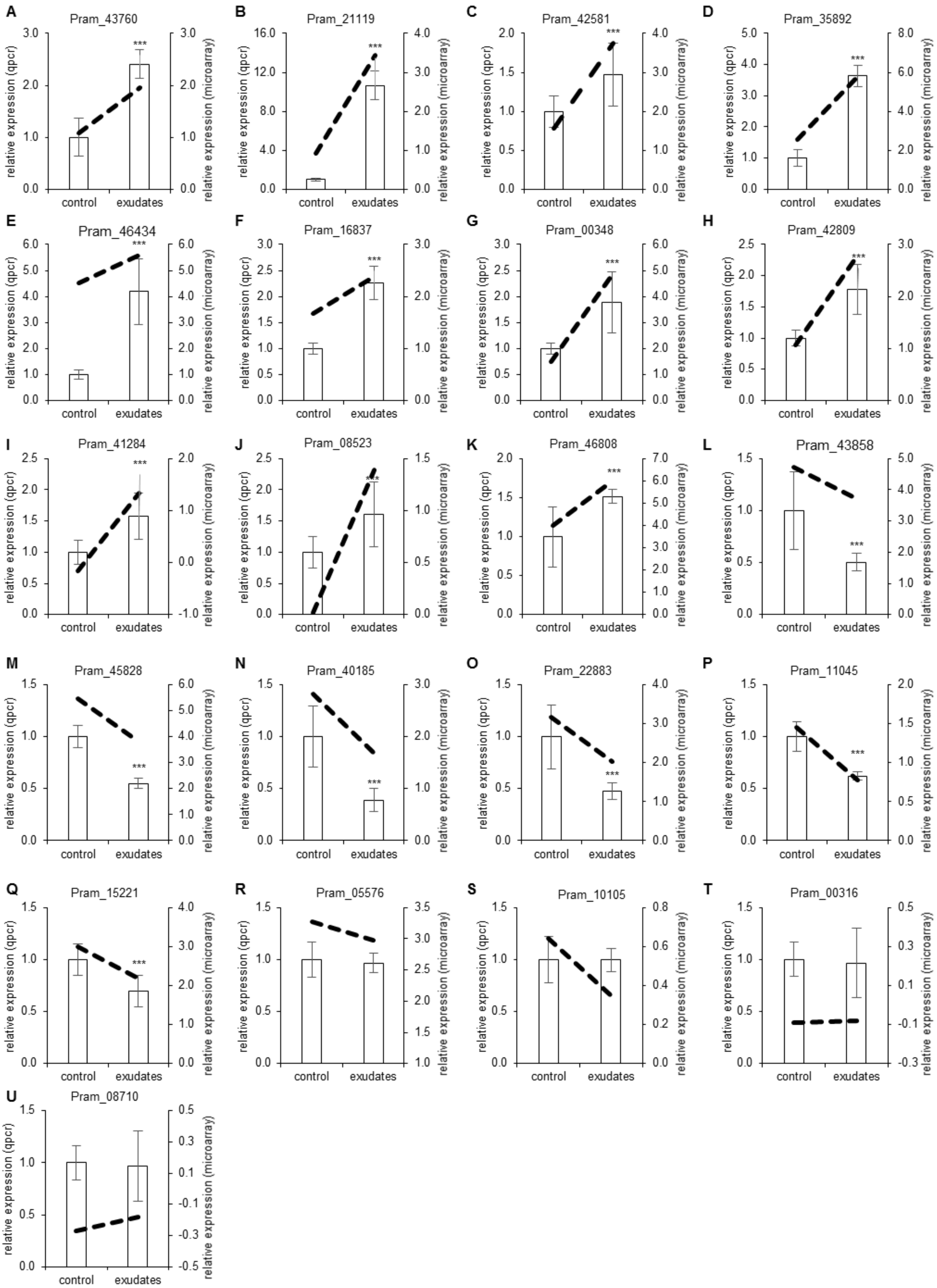

Figure S2: RT-qPCR validation of the expression profiles of selected genes.

Fold--induction of gene expression after *B. napus* root exudates or control (Coïc 50) treatment. For comparison, microarray data are shown in the graphs as dashed lines. Data are shown as log2 fold-

induction of treated germinated seeds compared to non-treated after 24 h of treatment. Two biological replicates were performed, each in three technical replicates. Means are values ± SD (n = 6). Means annotated with *** are significantly different from the control points (t-test, p <0.001).

Supplementary Data set 1: GO term enrichment analysis on *de novo* assembly of *P. ramosa* transcriptome (see joint Excel file)

Supplementary Data set 2: GO term enrichment analysis on the DEG set (see joint Excel file)
